# Epidural analgesia for the treatment of colic attack with retrocaval ureter in late pregnancy complicated with marginal placenta previa: a case report

**DOI:** 10.1186/s40981-019-0271-9

**Published:** 2019-08-14

**Authors:** Soshi Iwasaki, Kohsuke Hamada, Kazunobu Takahashi, Mika Takahashi, Eri Mizuno, Naomi Mizukami, Michiaki Yamakage

**Affiliations:** 1 Department of Anesthesiology, Sapporo Medical University, South1, West16, Chuo-ku, Sapporo City, Hokkaido Japan; 2 Ain Holdings and Nitori Holdings Department of Palliative Medicine, Sapporo Medical University, South1, West16, Chuo-ku, Sapporo City, Hokkaido Japan

**Keywords:** Retrocaval ureter, Hydronephrosis, Late pregnancy, Epidural analgesia, Placenta previa, Flank pain

## Abstract

**Background:**

Retrocaval ureter was diagnosed in a woman complaining of ureteric pain in the last trimester of pregnancy. We describe the rationale behind the administration of epidural analgesia for her colic attack.

**Case presentation:**

A 41-year-old pregnant woman was hospitalized with a diagnosis of a marginal placenta previa at 34 weeks and 5 days of pregnancy. Her right ureter encircled the dorsal aspect of the inferior vena cava (IVC) and was compressed by a growing fetus, causing hydronephrosis. Her right lower back pain was exacerbated every day, till an epidural catheter was inserted. Her estimated glomerular filtration rate (eGFR) and hematocrit worsened, and an elective cesarean section was performed.

**Conclusion:**

Epidural analgesia only provided pain relief for a few days. When a pregnant woman presents with a retrocaval ureter and severe pain, short-term epidural analgesia should be considered after evaluating the complex medical condition and size of the fetus.

## Background

Retrocaval ureter is a rare developmental anomaly, with an incidence of about 1 in 1000 births [1]. The ureter passes posterior to the inferior vena cava (IVC) and is asymptomatic till the third or fourth decade of life in most cases [2]. Some cases may be accidentally discovered in childhood during urological surgery [3] and when other congenital diseases [4] are present. The IVC compresses the ureter posteriorly, causing upstream dilatation of the proximal ureter and the kidney, resulting in flank pain, hematuria, pyelonephephritis, and urolithias [5]. It is a rare disease, with 2.8 to 3.0 times greater predominance [1, 3, 5] in males. Thus, reports of retrocaval ureters in pregnant women are extremely rare. Non-steroidal anti-inflammatory drugs (NSAIDs), or less effective opioids, such as morphine (when NSAIDs [6] are ineffective or contraindicated), are generally prescribed for acute flank pain in urological disease. However, NSAID administration in late pregnancy is associated with severe adverse neonatal outcomes [7]. No study in the literature describes an axial block for pain relief in pregnancy with retrocaval ureter. In this case, a woman presenting with ureteric pain due to hydronephrosis was diagnosed with retrocaval ureter in the last trimester of pregnancy, and she required continuous thoracic epidural analgesia. We describe the rationale behind the controversial decision to administer epidural analgesia for her colic attack.

### Case presentation

**Fig. 1:**
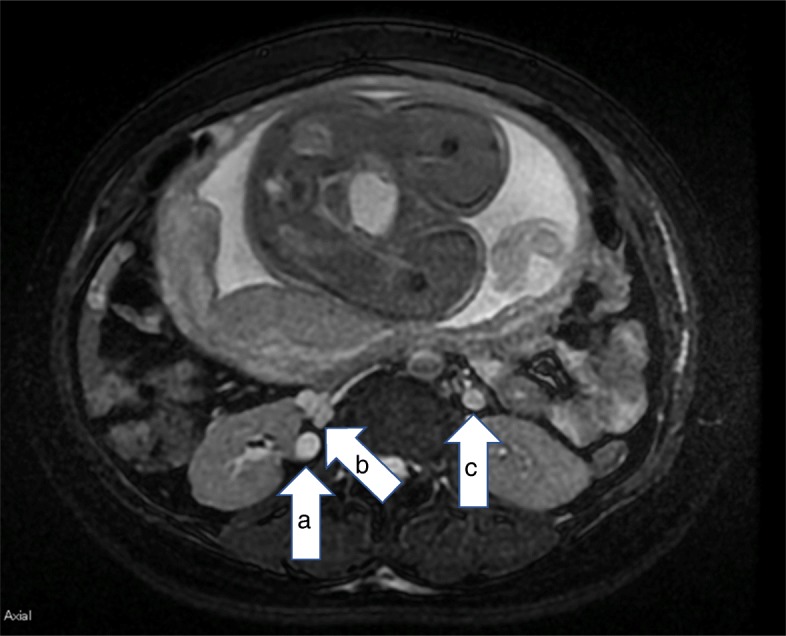
Axial abdominal T2-weighted MRI. **a** Dilated right ureter. **b** Inferior vena cava. **c** Left ureter. Legend: the right ureter (**a**) traveled to the dorsal side of the inferior vena cava (**b**) and was compressed by an enlarged fetus, causing hydronephrosis. The dilation of the left ureter (**c**) was less than that observed in **a** and is a physiological change seen in pregnant women

The patient was a 41-year-old woman, who was 34 weeks and 5 days pregnant. She had been hospitalized with a suspected diagnosis of placenta previa due to genital bleeding. Post-admission pelvic magnetic resonance imaging (MRI) revealed that the lowest end of the placenta was still in the internal cervix, i.e., a marginal placenta previa. Autologous blood donation was performed in preparation for a cesarean section, following which she began to complain of right lower back pain that worsened every day. Oral acetaminophen 1.8 g/day and intramuscular 15 mg pentazocine, administered every 3 h, were ineffective. As leukocyturia was observed, but urinary bacterial infection was not detected, the obstetrician suspected pyelonephritis, and a urologist was consulted. By reexamining the original MRI, the urologist determined that her right ureter encircled the dorsal aspect of the IVC and was being compressed by the growing fetus, causing hydronephrosis. The patient was diagnosed with right hydronephrosis due to retrocaval ureter (Fig. 1). He was not convinced regarding the efficacy of a urological stent in relieving pain of urological origin and preferred epidural analgesia. An elective cesarean section was planned by the obstetrician, who assembled a surgical team with sufficient personnel, including an interventional radiologist, keeping in mind the possibility of placenta previa. The department for pain control was apprised of her condition. Her hematocrit, leukocyte count, eGFR, and creatinine were 28.1% (reference range 33.5–45), 7000/μL (3500–9000), 93.1 mL/min/1.73m^2^, and 0.76 mL/dL (0.45–0.94), respectively, in the absence of coagulation disorders. We explained that epidural analgesia would not treat the stenosis, only provide pain relief, and the patient provided consent. With infusion of 1 g cefazolin, an epidural catheter was inserted at the T12–L1 interspace. Epidural analgesia was induced with 0.2% ropivacaine, at 4 mL/h, as the 4-mL bolus dose of 1% lidocaine was effective. Her NRS pain score decreased from 9 to 10 to 0 to 1 after epidural catheter insertion, and for 3 days no additional analgesia was needed. Her eGFR and hematocrit worsened to 69.7 mL/min/1.73 m^2^ and 26.8%, respectively, and an elective cesarean section, at 35 weeks of pregnancy, was performed. Ten milligrams of hyperbaric bupivacaine were administered for spinal analgesia, in addition to the insertion of the epidural catheter, with inter-departmental cooperation. Intraoperative hemorrhage, including amniotic fluid during surgery, was 1400 mL. Only autologous blood transfusion was required. The fetus weighed 2246 g at birth. Both mother and child were fine and were discharged within 2 weeks.

### Discussion and conclusion

Similar to an earlier report [3], dilation of the ureter by the IVC also occurred on the right side in our case. Due to compression of the right retrocaval ureter by the IVC and progressive compression by a growing fetus, the pain relief provided by epidural analgesia only lasted for a few days. Failure to alleviate severe pain in a pregnant woman exposes the fetus to undesirable effects due to cortisol exposure [8]. However, abdominal muscle relaxation due to epidural analgesia may itself aggravate compression, as the pressure from the front to the back of the fetus may cause supine hypotension syndrome, which is well known to anesthesiologists.

Surgical management of placenta previa is difficult because of high maternal morbidity and frequent [9] neonatal complications. Interventional radiology using autologous blood can help control hemorrhage in pregnancies complicated by placenta accreta [10]. Known contraindications for collecting autologous blood include infection, antibiotic use, preoperative anemia, malignancy, and sickle cell disease [11]; none of these contraindications were present in our patient so we proceeded with the blood collection. However, our patient experienced pain immediately following the autologous blood collection, and it is possible that IVC collapse aggravated her condition. This suggests that clinicians should carefully weigh the risk of autologous blood collection causing pain in patients with diagnosed retrocaval ureter.

As evidenced by this case, epidural analgesia may help to gain time for planning the surgery and for anesthetic management thereafter. Surely, this case was rare. It may repeatedly occur in pregnant women because of the frequency prescribed and mechanism of onset being easy to understand. In this case, from the expiration date of autologous blood transfusion, we expected epidural catheter placement for about a week. There have been reports of epidural abscess in pregnant women [12], and it is evident that long-term placement increases the risk of epidural abscess. Pain management using epidural anesthesia in the retrocaval ureter in pregnant women should be used to prepare for cesarean section in a short time. Fortunately, severe pain like that described in this case is likely to occur later in pregnancy. The fetus at 35 weeks of gestation is estimated to weigh about 2500 g [13], and depending on the health condition of the mother, cesarean section is indicated. If there are no symptoms, conservative management is possible while monitoring renal function and infection [14].

Moreover, pyelonephritis [15] and ureteral calculi [16] are not uncommon during pregnancy (their incidence during pregnancy is estimated to be 1–2% and 0.02–0.53%, respectively); therefore, pelvic MRI is essential for the differentiation of pain. We did not consider that this case was successfully managed, but when a retrocaval ureter causes severe pain during pregnancy, the size of the fetus and the complex nature of the mother’s condition should be taken into account and short-term epidural analgesia should be considered, with careful clinical observation.

## Data Availability

Not applicable.

## Abbreviations

CRP: C-reactive protein
CT: Computed tomography
eGFR: Estimated glomerular filtration rate
IVC: Infra vena cava
MRI: Magnetic resonance imaging
NRS: Numerical rating scale
WBC: White blood cell

## References

[1] de Arruda GJF, de Arruda Neto JF, Eroles JC, Spessoto LCF, de Arruda JGF, Fácio FN (2018). Incidental finding of retrocaval ureter in a patient without hydronephrosis. AME Case Rep.

[2] Kyei MY, Yeboah ED, Klufio GO, Mensah JE, Gepi-Atee S, Zakpaa L, Morton B, Adusei B (2011). Retrocaval ureter: two case reports. Ghana Med J.

[3] Junejo NN, Vallasciani S, Peters C, AlHazmi H, Almathami A, Alshammari A, AlJallad H, Azar F, Abasher A, AlShahrani S (2018). High retrocaval ureter: an unexpected intraoperative finding during robotic redo pyeloplasty. Urol Case Rep.

[4] Fernando MH, Jayarajah U, Arulanantham A, Goonewardena S, Wijewardena M (2018). Retrocaval ureter associated with cryptorchidism: a case report and review of literature. Clin Case Rep.

[5] Guttilla A, Fiorello M, Fulcoli V, Andrisano A, Massari D, Costa G (2018). A case of retrograde treatment of a ureteral stone in a retrocaval ureter. J Endourol Case Rep.

[6] Pathan Sameer A., Mitra Biswadev, Romero Lorena, Cameron Peter A. (2017). What is the best analgesic option for patients presenting with renal colic to the emergency department? Protocol for a systematic review and meta-analysis. BMJ Open.

[7] Damase-Michel C, Christaud J, Berrebi A, Lacroix I, Montastruc JL (2009). What do pregnant women know about non-steroidal anti-inflammatory drugs?. c Drug Saf.

[8] O’Donnell KJ, Bugge Jensen A, Freeman L, Khalife N, O’Connor TG, Glover V (2012). Maternal prenatal anxiety and downregulation of placental 11β-HSD2. Psychoneuroendocrinology..

[9] Kollmann M, Gaulhofer J, Lang U, Klaritsch P (2016). Placenta praevia: incidence, risk factors and outcome. J Matern Fetal Neonatal Med.

[10] Duan X, Chen P, Han X, Wang Y, Chen Z, Zhang X, Chu Q, Liang H (2018). Intermittent aortic balloon occlusion combined with cesarean section for the treatment of patients with placenta previa complicated by placenta accreta: a retrospective study. J Obstet Gynaecol Res.

[11] Ćatić D, Milojković A, Steblovnik L (2018). Preoperative autologous blood donation in placenta previa patients. Transfus Apher Sci.

[12] Tumber SS, Liu H (2010). Epidural abscess after multiple lumbar punctures for labour epidural catheter placement. J Biomed Res.

[13] Salomon LJ, Bernard JP, Ville Y (2007). Estimation of fetal weight: reference range at 20-36 weeks' gestation and comparison with actual birth-weight reference range. Ultrasound Obstet Gynecol.

[14] Yen JM, Lee LS, Cheng CW (2015). Conservative management of retrocaval ureter: a case series. Int J Surg Case Rep.

[15] Nien JK, Romero R, Hoppensteadt D, Erez O, Espinoza J, Soto E, Kusanovic JP, Gotsch F, Kim CJ, Mittal P, Fareed J, Santolaya J, Chaiworapongsa T, Edwin S, Pineles B, Hassan S (2008). Pyelonephritis during pregnancy: a cause for an acquired deficiency of protein Z. J Matern Fetal Neonatal Med.

[16] Butticè S, Laganà AS, Vitale SG, Netsch C, Tanidir Y, Cantiello F, Dragos L, Talso M, Emiliani E, Pappalardo R, Sener TE (2017). Ureteroscopy in pregnant women with complicated colic pain: is there any risk of premature labor?. Arch Ital Urol Androl.

